# Diethyl 4,4′-dihy­droxy-3,3′-{[(3a*RS*,7a*RS*)-2,3,3a,4,5,6,7,7a-octa­hydro-1*H*-1,3-benzimidazole-1,3-di­yl]bis­(methyl­ene)}dibenzoate

**DOI:** 10.1107/S1600536811039559

**Published:** 2011-10-05

**Authors:** Augusto Rivera, Diego Quiroga, Jaime Ríos-Motta, Karla Fejfarová, Michal Dušek

**Affiliations:** a Departamento de Química, Universidad Nacional de Colombia, Ciudad Universitaria, Bogotá, Colombia; b Institute of Physics ASCR, v.v.i., Na Slovance 2, 182 21 Praha 8, Czech Republic

## Abstract

The heterocyclic ring in the title compound, C_27_H_34_N_2_O_6_, has an envelope conformation on one of the bridgehead C atoms [*Q*(2) = 0.4487 (19) Å and ϕ = 291.3 (2)°]. Two strong intra­molecular O—H⋯N hydrogen bonds stabilize the mol­ecular conformation. The benzoate groups differ in the relative orientations of the ethyl groups, as quanti­fied by the values of the C—O—C—C torsion angles of −86.5 (2) and −178.97 (17)°. The carbonyl groups are nearly coplanar with the benzene rings, forming C—C—C—O torsion angles of 0.9 (3) and 3.4 (3)°. The crystal structure is stabilized by weak inter­molecular C—H⋯O inter­actions.

## Related literature

For related structures, see: Rivera *et al.* (2010 ▶, 2011**a* ▶,b* ▶). For the background to this work, see: Van den Enden & Geise (1981 ▶); Geise *et al.* (1971 ▶). For the synthesis of the precursor, see: Murray-Rust & Riddell (1975 ▶). For puckering parameters, see: Cremer & Pople (1975 ▶). For hydrogen-bond graph-set nomenclature, see: Bernstein *et al.* (1995 ▶).
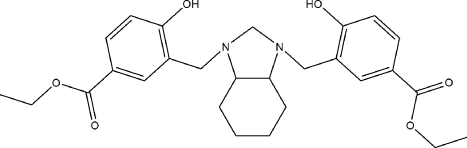


## Experimental

### 

#### Crystal data

C_27_H_34_N_2_O_6_
*M* *_r_* = 482.6
Triclinic, 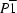

*a* = 8.1132 (4) Å
*b* = 10.9796 (7) Å
*c* = 15.2450 (8) Å
α = 89.580 (5)°
β = 81.028 (4)°
γ = 70.028 (5)°
*V* = 1259.19 (13) Å^3^
*Z* = 2
Cu *K*α radiation
μ = 0.73 mm^−1^
*T* = 120 K
0.46 × 0.18 × 0.11 mm

#### Data collection

Agilent Xcalibur diffractometer with an Atlas (Gemini ultra Cu) detector
Absorption correction: multi-scan (*CrysAlis PRO*; Agilent, 2010 ▶) *T*_min_ = 0.752, *T*_max_ = 1
11294 measured reflections
4430 independent reflections
3313 reflections with *I* > 3σ(*I*)
*R*_int_ = 0.034

#### Refinement

*R*[*F* ^2^ > 2σ(*F* ^2^)] = 0.043
*wR*(*F* ^2^) = 0.111
*S* = 1.64
4430 reflections
323 parameters
H atoms treated by a mixture of independent and constrained refinement
Δρ_max_ = 0.47 e Å^−3^
Δρ_min_ = −0.24 e Å^−3^

### 

Data collection: *CrysAlis PRO* (Agilent, 2010 ▶); cell refinement: *CrysAlis PRO*; data reduction: *CrysAlis PRO*; program(s) used to solve structure: *SIR2002* (Burla *et al.*, 2003 ▶); program(s) used to refine structure: *JANA2006* (Petříček *et al.*, 2006 ▶); molecular graphics: *DIAMOND* (Brandenburg & Putz, 2005 ▶); software used to prepare material for publication: *JANA2006*.

## Supplementary Material

Crystal structure: contains datablock(s) global, I. DOI: 10.1107/S1600536811039559/bt5654sup1.cif

Structure factors: contains datablock(s) I. DOI: 10.1107/S1600536811039559/bt5654Isup2.hkl

Supplementary material file. DOI: 10.1107/S1600536811039559/bt5654Isup3.cml

Additional supplementary materials:  crystallographic information; 3D view; checkCIF report

## Figures and Tables

**Table 1:** Hydrogen-bond geometry (Å, °)

*D*—H⋯*A*	*D*—H	H⋯*A*	*D*⋯*A*	*D*—H⋯*A*
O3—H3*o*⋯N1	0.89 (3)	1.82 (3)	2.663 (2)	156.9 (19)
O6—H6*o*⋯N2	0.91 (2)	1.82 (3)	2.669 (2)	154 (3)
C2—H2⋯O1^i^	0.96	2.58	3.362 (2)	138
C3—H3⋯O4^ii^	0.96	2.57	3.436 (2)	151
C8—H8*b*⋯O1^i^	0.96	2.57	3.336 (2)	137
C22—H22⋯O3^iii^	0.96	2.44	3.351 (2)	159

Symmetry codes: (i) 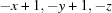; (ii) 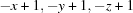; (iii) 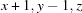.

**Fig. 1.**
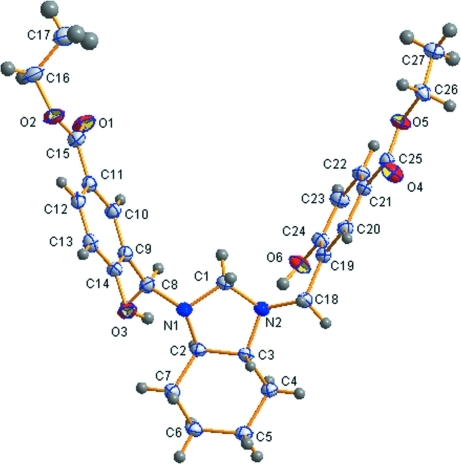
A view of (I) with the numbering scheme. Displacement ellipsoids are drawn at the 50% probability level.

**Fig. 2.**
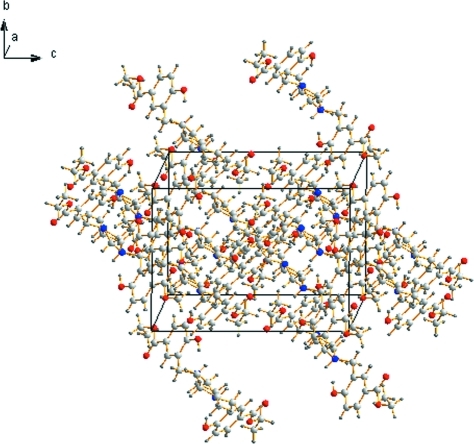
Packing of the molecules of the title compound view along the a axis.

**Fig. 3.**
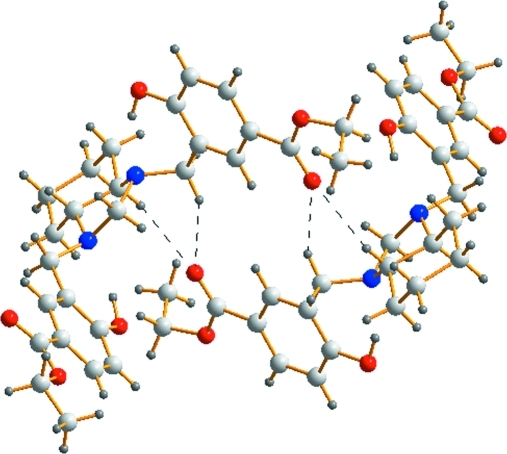
Dimer formation of the title compound by a R22(14) ring motif.

## supplementary crystallographic information

### Comment

The title compound (I) was obtained from ethyl *p*-hydroxybenzoate and (2*R*,7*R*,11*S*,16*S*)-1,8,10,17- tetraazapentacyclo[8.8.1.1^8,17^.0^2,7^.0^11,16^]icosane by a Mannich type reaction as an extension of our work on the structural studies of *di*-Mannich bases with the 2,3,3a,4,5,6,7,7a-octahydro-1*H*-1,3-benzimidazole chiral core (Rivera *et al.*, 2010; Rivera *et al.*, 2011**a*,b*).

In the molecule of the title compound (Fig. 1), *x*-rays analysis indicated that in the cyclohexane ring the C7—C2—C3—C4 endocyclic torsion angle is increased from the normal 55° to 65.6 (2) °. The endocyclic N1—C2—C3—N2 torsion angle in the heterocyclic ring is -45.57 (16) °, which is in the order of the maximum value for torsion angles in five-membered rings (Van den Enden & Geise, 1981). These results confirm the existence of a puckering of the perhydrobenzimidazole moiety, where the 1,2-cyclohexanediamine fragment adopts a chair conformation with shorter endocyclic bond angles [C3—C4—C5, 106.90 (17)°; C2—C7—C6, 106.70 (17)°] and longer bond angles [C4—C5—C6, 112.75 (15); C5—C6—C7; 112.22 (17)°] respect to the normal bond angles [111.4 °. Geise *et al.*, 1971] in a ideal chair conformation. The heterocyclic ring has a envelope conformation on C3 (Q(2) = 0.4487 (19) Å, φ = 291.3 (2)°) (Cremer & Pople, 1975) with endocyclic bond angles between 100.86 (13)° and 106.30 (15)° which are shorter respect the tetrahedrical normal bond angles.

The benzoate moieties differ in the relative orientations of the ethyl groups, Fig. 1, as quantified in the values of the C15—O2—C16—C17 and C25—O5—C26—C27 torsion angles of -86.5 (2) and -178.97 (17)°, respectively, which indicate different orientations with respect to the plane of benzoate moiety. The carbonyl groups are nearly coplanar with the benzene rings forming C10—C11—C15—O1 and C20—C21—C25—O4 torsion angles of 0.9 (3)° and 3.4 (3)° respectively. Bond angles around the carbonyl C atom deviate slightly from 120°. The C—C bond lengths between the atoms in the sequences C9, C10 and C12, C13, [1.385 (2) and 1.382 (2) Å] respectively are similar whereas the C10—C11 [1.400 (3) Å] and C11—C12 [1.392 (3) Å], bond lengths are slightly longer because of the influence of the polar C═O group while the C9—C14 [1.359 (2) Å] is shorter and C13—C14 [1.392 (3) Å] is slightly longer because of the influence of the O—H hydrogen bonded groups.

In the crystal, adjacent molecules are connected *via* intermolecular C—H···O hydrogen bonds, forming an one-dimensional chain which propagates parallel with the *c* axis (Fig. 2) where one intermolecular hydrogen-bonding *R*^2^_2_ (14) (Bernstein *et al.* 1995) graph-set motifs is generated (Fig 3).

### Experimental

To a dioxane:water (7 ml) solution of the aminal (2*R*,7*R*,11*S*,16*S*)-1,8,10,17-tetraazapentacyclo[8.8.1.1^8,17^.0^2,7^.0^11,16^]icosane (276 mg, 1.00 mmol) prepared previously following described procedures (Murray-Rust & Riddell, 1975), was added dropwise a dioxane solution (3 ml) containing two equivalents of ethyl *p*-hydroxybenzoate (332 mg, 2.00 mmol). The mixture was refluxed for about 10 h. The solvent was evaporated under reduced pressure until a sticky residue appeared. The product was purified by chromatography on a silica column, and subjected to gradient elution with benzene:ethyl acetate (yield 18%, m.p. = 408–410 K). Single crystals of racemic (I) were grown from a chloroform: methanol solution by slow evaporation of the solvent at room temperature over a period of about 2 weeks.

^1^H NMR (CDCl_3_, 400 MHz): δ 1.28 (4*H*, m), 1.35 (6*H*, t, ^3^*J*_H,H_ = 7.2 Hz), 1.86 (2*H*, m), 2.07 (2*H*, m), 2.40 (2*H*, m), 3.56 (2*H*, d, ^2^*J*_H,H_ = 13.9 Hz, ArCH_2_N), 3.56 (2*H*, s, NCH_2_N), 4.20 (2*H*, d, ^2^*J*_H,H_ = 13.9 Hz, ArCH_2_N), 4.30 (4*H*, q, ^3^*J*_H,H_ = 7.2 Hz), 6.83 (2*H*, d, ^3^*J*_H,H_ = 8.5 Hz), 7.68 (2*H*, d, ^4^*J*_H,H_ = 2.2 Hz), 7.87 (2*H*, dd, ^3^*J*_H,H_ = 8.5 Hz, ^4^*J*_H,H_ = 2.2 Hz). ^13^C NMR (CDCl_3_, 100 MHz): δ 14.4, 23.9, 28.9, 56.0, 60.6, 69.1, 75.6, 116.2, 121.0, 121.8, 130.0, 131.2, 161.8, 166.3.

### Refinement

All hydrogen atoms were discernible in difference Fourier maps and could be refined to reasonable geometry. According to common practice H atoms bonded C atoms were kept in ideal positions with C–H distance 0.96 Å during the refinement. The methyl H atoms were allowed to rotate freely about the adjacent C—C bonds. The hydroxyl H atoms were found in difference Fourier maps and their coordinates were refined freely. All H atoms were refined with displacement displacement coefficients *U*_iso_(H) set to 1.5Ueq(C, O) for methyl and hydroxyl groups and to to 1.2Ueq(C) for the CH– and CH_2_- groups.

### Crystal data

**Table:**

C_27_H_34_N_2_O_6_	*Z* = 2
*M**_r_* = 482.6	*F*(000) = 516
Triclinic, *P*1	*D*_x_ = 1.272 Mg m^−^^3^
Hall symbol: -P 1	Cu *K*α radiation, λ = 1.5418 Å
*a* = 8.1132 (4) Å	Cell parameters from 4486 reflections
*b* = 10.9796 (7) Å	θ = 2.9–67.1°
*c* = 15.2450 (8) Å	µ = 0.73 mm^−^^1^
α = 89.580 (5)°	*T* = 120 K
β = 81.028 (4)°	Block, colourless
γ = 70.028 (5)°	0.46 × 0.18 × 0.11 mm
*V* = 1259.19 (13) Å^3^	

### Data collection

**Table:**

Agilent Xcalibur diffractometer with an Atlas (Gemini ultra Cu) detector	4430 independent reflections
Radiation source: Enhance Ultra (Cu) X-ray Source	3313 reflections with *I* > 3σ(*I*)
mirror	*R*_int_ = 0.034
Detector resolution: 10.3784 pixels mm^-1^	θ_max_ = 67.2°, θ_min_ = 2.9°
Rotation method data acquisition using ω scans	*h* = −8→9
Absorption correction: multi-scan (*CrysAlis PRO*; Agilent, 2010)	*k* = −13→12
*T*_min_ = 0.752, *T*_max_ = 1	*l* = −18→18
11294 measured reflections	

### Refinement

**Table:**

Refinement on *F*^2^	H atoms treated by a mixture of independent and constrained refinement
*R*[*F*^2^ > 2σ(*F*^2^)] = 0.043	Weighting scheme based on measured s.u.'s *w* = 1/[σ^2^(*I*) + 0.0009*I*^2^]
*wR*(*F*^2^) = 0.111	(Δ/σ)_max_ = 0.005
*S* = 1.64	Δρ_max_ = 0.47 e Å^−^^3^
4430 reflections	Δρ_min_ = −0.24 e Å^−^^3^
323 parameters	Extinction correction: B-C type 1 Lorentzian isotropic (Becker & Coppens, 1974)
0 restraints	Extinction coefficient: 900 (200)
130 constraints	

### Special details

**Table:**

Experimental. CrysAlisPro (Agilent Technologies, 2010) Empirical absorption correction using spherical harmonics, implemented in SCALE3 ABSPACK scaling algorithm
Refinement. The refinement was carried out against all reflections. The conventional *R*-factor is always based on *F*. The goodness of fit as well as the weighted *R*-factor are based on *F* and *F*^2^ for refinement carried out on *F* and *F*^2^, respectively. The threshold expression is used only for calculating *R*-factors *etc*. and it is not relevant to the choice of reflections for refinement.The program used for refinement, Jana2006, uses the weighting scheme based on the experimental expectations, see _refine_ls_weighting_details, that does not force *S* to be one. Therefore the values of *S* are usually larger than the ones from the *SHELX* program.

### Fractional atomic coordinates and isotropic or equivalent isotropic displacement parameters (Å^2^)

**Table:**

	*x*	*y*	*z*	*U*_iso_*/*U*_eq_	
O1	0.80490 (18)	0.62458 (15)	−0.09060 (10)	0.0383 (6)	
O2	0.86246 (17)	0.80304 (15)	−0.05677 (10)	0.0363 (6)	
O3	0.16819 (17)	0.90030 (14)	0.22196 (9)	0.0305 (5)	
O4	0.88617 (18)	0.26784 (14)	0.50761 (10)	0.0336 (5)	
O5	1.05002 (16)	0.09728 (14)	0.41510 (9)	0.0289 (5)	
O6	0.35533 (18)	0.23291 (15)	0.24830 (10)	0.0327 (6)	
N1	0.16240 (18)	0.65935 (16)	0.21904 (10)	0.0230 (6)	
N2	0.20550 (18)	0.47944 (15)	0.31013 (10)	0.0223 (5)	
C1	0.2970 (2)	0.5617 (2)	0.26184 (14)	0.0301 (7)	
C2	−0.0010 (2)	0.62702 (18)	0.23985 (12)	0.0215 (6)	
C3	0.0186 (2)	0.56664 (18)	0.32802 (12)	0.0212 (6)	
C4	−0.1214 (2)	0.5040 (2)	0.35509 (13)	0.0251 (7)	
C5	−0.3031 (2)	0.6115 (2)	0.36049 (13)	0.0284 (7)	
C6	−0.3246 (2)	0.6841 (2)	0.27465 (13)	0.0289 (7)	
C7	−0.1753 (2)	0.7398 (2)	0.24635 (13)	0.0263 (7)	
C8	0.2195 (2)	0.66840 (19)	0.12354 (13)	0.0247 (7)	
C9	0.3493 (2)	0.74075 (18)	0.10787 (12)	0.0220 (6)	
C10	0.5003 (2)	0.69903 (19)	0.04363 (13)	0.0237 (7)	
C11	0.6112 (2)	0.77285 (19)	0.02585 (12)	0.0237 (7)	
C12	0.5683 (2)	0.89060 (19)	0.07296 (12)	0.0243 (7)	
C13	0.4197 (2)	0.93306 (19)	0.13820 (13)	0.0252 (7)	
C14	0.3118 (2)	0.85782 (19)	0.15638 (12)	0.0235 (7)	
C15	0.7677 (2)	0.7236 (2)	−0.04577 (13)	0.0281 (7)	
C16	1.0021 (3)	0.7736 (2)	−0.13485 (16)	0.0406 (9)	
C17	1.1722 (3)	0.6775 (3)	−0.11692 (17)	0.0510 (11)	
C18	0.2807 (2)	0.42669 (18)	0.38978 (13)	0.0234 (6)	
C19	0.4584 (2)	0.31872 (18)	0.36541 (12)	0.0216 (6)	
C20	0.5969 (2)	0.30589 (19)	0.41223 (12)	0.0226 (6)	
C21	0.7585 (2)	0.20349 (18)	0.39261 (12)	0.0223 (6)	
C22	0.7816 (2)	0.11224 (19)	0.32436 (13)	0.0248 (7)	
C23	0.6454 (2)	0.1236 (2)	0.27718 (13)	0.0275 (7)	
C24	0.4850 (2)	0.22611 (19)	0.29689 (13)	0.0239 (7)	
C25	0.9003 (2)	0.19539 (18)	0.44524 (13)	0.0233 (7)	
C26	1.2010 (2)	0.0805 (2)	0.45986 (14)	0.0289 (7)	
C27	1.3504 (2)	−0.0335 (2)	0.41217 (14)	0.0335 (8)	
H1a	0.390996	0.509476	0.217022	0.0361*	
H1b	0.339167	0.60455	0.303387	0.0361*	
H2	−0.009721	0.573115	0.192756	0.0257*	
H3	−0.002172	0.624178	0.378853	0.0255*	
H4a	−0.109705	0.438854	0.310572	0.0301*	
H4b	−0.109034	0.468638	0.412473	0.0301*	
H5a	−0.395371	0.574881	0.374116	0.034*	
H5b	−0.319889	0.671905	0.409118	0.034*	
H6a	−0.326041	0.626526	0.227901	0.0346*	
H6b	−0.437513	0.753203	0.282548	0.0346*	
H7a	−0.186338	0.775993	0.189146	0.0316*	
H7b	−0.180183	0.802846	0.290901	0.0316*	
H8a	0.117525	0.712073	0.096222	0.0296*	
H8b	0.274173	0.582741	0.095879	0.0296*	
H10	0.529284	0.618178	0.010754	0.0285*	
H12	0.642366	0.942596	0.060072	0.0292*	
H13	0.390889	1.014069	0.170841	0.0302*	
H16a	0.963679	0.740749	−0.183166	0.0488*	
H16b	1.021028	0.852032	−0.153732	0.0488*	
H17a	1.262112	0.665384	−0.168345	0.0765*	
H17b	1.208207	0.708856	−0.067132	0.0765*	
H17c	1.156002	0.596333	−0.103694	0.0765*	
H18a	0.199541	0.394252	0.426876	0.0281*	
H18b	0.295301	0.494797	0.423447	0.0281*	
H20	0.581029	0.368816	0.459072	0.0271*	
H22	0.892295	0.041523	0.310208	0.0298*	
H23	0.66159	0.060322	0.230516	0.033*	
H26a	1.172541	0.060967	0.520634	0.0347*	
H26b	1.233698	0.156681	0.454438	0.0347*	
H27a	1.31469	−0.108441	0.414248	0.0503*	
H27b	1.378537	−0.014112	0.351344	0.0503*	
H27c	1.453165	−0.050579	0.440505	0.0503*	
H3o	0.139 (3)	0.829 (3)	0.2311 (15)	0.0366*	
H6o	0.280 (3)	0.316 (3)	0.2587 (16)	0.0393*	

### Atomic displacement parameters (Å^2^)

**Table:**

	*U*^11^	*U*^22^	*U*^33^	*U*^12^	*U*^13^	*U*^23^
O1	0.0355 (8)	0.0382 (9)	0.0379 (9)	−0.0156 (7)	0.0104 (6)	−0.0086 (7)
O2	0.0280 (7)	0.0358 (9)	0.0439 (9)	−0.0156 (6)	0.0079 (6)	−0.0004 (7)
O3	0.0272 (7)	0.0266 (8)	0.0324 (8)	−0.0073 (6)	0.0060 (6)	−0.0034 (6)
O4	0.0325 (8)	0.0300 (8)	0.0357 (8)	−0.0042 (6)	−0.0127 (6)	−0.0063 (7)
O5	0.0199 (7)	0.0305 (8)	0.0329 (8)	−0.0025 (6)	−0.0080 (5)	−0.0027 (6)
O6	0.0278 (7)	0.0283 (8)	0.0416 (9)	−0.0046 (6)	−0.0155 (6)	−0.0064 (7)
N1	0.0175 (7)	0.0280 (9)	0.0235 (8)	−0.0080 (6)	−0.0031 (6)	0.0047 (7)
N2	0.0173 (7)	0.0239 (9)	0.0238 (8)	−0.0047 (6)	−0.0034 (6)	0.0040 (7)
C1	0.0193 (9)	0.0345 (12)	0.0359 (12)	−0.0084 (8)	−0.0055 (8)	0.0115 (9)
C2	0.0175 (9)	0.0246 (10)	0.0230 (10)	−0.0082 (7)	−0.0035 (7)	0.0022 (8)
C3	0.0174 (9)	0.0221 (10)	0.0216 (9)	−0.0039 (7)	−0.0024 (7)	−0.0009 (8)
C4	0.0203 (9)	0.0296 (11)	0.0262 (10)	−0.0101 (8)	−0.0027 (7)	0.0042 (8)
C5	0.0193 (9)	0.0357 (12)	0.0294 (11)	−0.0098 (8)	−0.0015 (8)	0.0025 (9)
C6	0.0173 (9)	0.0362 (12)	0.0305 (11)	−0.0058 (8)	−0.0044 (8)	0.0024 (9)
C7	0.0216 (9)	0.0290 (11)	0.0255 (10)	−0.0048 (8)	−0.0042 (8)	0.0045 (8)
C8	0.0244 (9)	0.0255 (10)	0.0230 (10)	−0.0089 (8)	0.0002 (7)	0.0004 (8)
C9	0.0223 (9)	0.0228 (10)	0.0209 (9)	−0.0077 (7)	−0.0041 (7)	0.0038 (8)
C10	0.0247 (9)	0.0232 (10)	0.0224 (10)	−0.0073 (8)	−0.0034 (7)	0.0012 (8)
C11	0.0229 (9)	0.0273 (10)	0.0214 (10)	−0.0091 (8)	−0.0044 (7)	0.0033 (8)
C12	0.0258 (10)	0.0250 (10)	0.0250 (10)	−0.0108 (8)	−0.0081 (8)	0.0068 (8)
C13	0.0284 (10)	0.0218 (10)	0.0253 (10)	−0.0075 (8)	−0.0073 (8)	0.0015 (8)
C14	0.0217 (9)	0.0226 (10)	0.0222 (10)	−0.0032 (7)	−0.0021 (7)	0.0026 (8)
C15	0.0262 (10)	0.0305 (11)	0.0283 (11)	−0.0117 (8)	−0.0022 (8)	0.0041 (9)
C16	0.0318 (11)	0.0473 (14)	0.0405 (13)	−0.0171 (10)	0.0093 (9)	0.0016 (11)
C17	0.0365 (13)	0.0683 (19)	0.0445 (15)	−0.0167 (12)	0.0008 (11)	0.0037 (13)
C18	0.0220 (9)	0.0233 (10)	0.0226 (10)	−0.0051 (7)	−0.0032 (7)	0.0020 (8)
C19	0.0211 (9)	0.0209 (10)	0.0222 (9)	−0.0066 (7)	−0.0027 (7)	0.0030 (8)
C20	0.0239 (9)	0.0214 (10)	0.0209 (9)	−0.0071 (7)	−0.0010 (7)	0.0000 (8)
C21	0.0219 (9)	0.0218 (10)	0.0226 (10)	−0.0075 (7)	−0.0018 (7)	0.0038 (8)
C22	0.0208 (9)	0.0226 (10)	0.0275 (10)	−0.0039 (8)	−0.0017 (8)	−0.0013 (8)
C23	0.0273 (10)	0.0247 (11)	0.0288 (11)	−0.0070 (8)	−0.0038 (8)	−0.0049 (8)
C24	0.0219 (9)	0.0234 (10)	0.0279 (10)	−0.0089 (8)	−0.0064 (8)	0.0010 (8)
C25	0.0229 (9)	0.0207 (10)	0.0256 (10)	−0.0069 (8)	−0.0037 (8)	0.0032 (8)
C26	0.0235 (10)	0.0298 (11)	0.0353 (11)	−0.0081 (8)	−0.0130 (8)	0.0056 (9)
C27	0.0217 (10)	0.0369 (12)	0.0396 (12)	−0.0064 (8)	−0.0067 (9)	0.0036 (10)

### Geometric parameters (Å, °)

**Table:**

O1—C15	1.209 (3)	C8—H8b	0.96
O2—C15	1.340 (3)	C9—C10	1.385 (2)
O2—C16	1.463 (3)	C9—C14	1.403 (3)
O3—C14	1.359 (2)	C10—C11	1.400 (3)
O3—H3o	0.89 (3)	C10—H10	0.96
O4—C25	1.210 (3)	C11—C12	1.392 (3)
O5—C25	1.3364 (19)	C11—C15	1.485 (2)
O5—C26	1.451 (3)	C12—C13	1.382 (2)
O6—C24	1.360 (3)	C12—H12	0.96
O6—H6o	0.91 (2)	C13—C14	1.393 (3)
N1—C1	1.476 (2)	C13—H13	0.96
N1—C2	1.474 (3)	C16—C17	1.487 (3)
N1—C8	1.471 (2)	C16—H16a	0.96
N2—C1	1.479 (3)	C16—H16b	0.96
N2—C3	1.474 (2)	C17—H17a	0.96
N2—C18	1.470 (2)	C17—H17b	0.96
C1—H1a	0.96	C17—H17c	0.96
C1—H1b	0.96	C18—C19	1.514 (2)
C2—C3	1.501 (3)	C18—H18a	0.96
C2—C7	1.518 (2)	C18—H18b	0.96
C2—H2	0.96	C19—C20	1.390 (3)
C3—C4	1.522 (3)	C19—C24	1.402 (3)
C3—H3	0.96	C20—C21	1.396 (2)
C4—C5	1.533 (2)	C20—H20	0.96
C4—H4a	0.96	C21—C22	1.396 (3)
C4—H4b	0.96	C21—C25	1.481 (3)
C5—C6	1.529 (3)	C22—C23	1.380 (3)
C5—H5a	0.96	C22—H22	0.96
C5—H5b	0.96	C23—C24	1.391 (2)
C6—C7	1.539 (3)	C23—H23	0.96
C6—H6a	0.96	C26—C27	1.505 (2)
C6—H6b	0.96	C26—H26a	0.96
C7—H7a	0.96	C26—H26b	0.96
C7—H7b	0.96	C27—H27a	0.96
C8—C9	1.512 (3)	C27—H27b	0.96
C8—H8a	0.96	C27—H27c	0.96
			
C15—O2—C16	116.06 (17)	C10—C11—C15	117.93 (18)
C14—O3—H3o	102.3 (13)	C12—C11—C15	122.6 (2)
C25—O5—C26	117.08 (16)	C11—C12—C13	120.4 (2)
C24—O6—H6o	103.4 (18)	C11—C12—H12	119.8207
C1—N1—C2	105.79 (16)	C13—C12—H12	119.8195
C1—N1—C8	113.67 (13)	C12—C13—C14	119.71 (19)
C2—N1—C8	114.09 (16)	C12—C13—H13	120.1478
C1—N2—C3	102.83 (14)	C14—C13—H13	120.1472
C1—N2—C18	112.75 (16)	O3—C14—C9	120.36 (19)
C3—N2—C18	114.62 (13)	O3—C14—C13	118.70 (18)
N1—C1—N2	106.30 (15)	C9—C14—C13	120.94 (16)
N1—C1—H1a	109.4712	O1—C15—O2	123.33 (17)
N1—C1—H1b	109.4709	O1—C15—C11	124.4 (2)
N2—C1—H1a	109.4716	O2—C15—C11	112.25 (18)
N2—C1—H1b	109.4715	O2—C16—C17	112.13 (19)
H1a—C1—H1b	112.4688	O2—C16—H16a	109.4709
N1—C2—C3	101.91 (15)	O2—C16—H16b	109.4712
N1—C2—C7	116.25 (17)	C17—C16—H16a	109.4713
N1—C2—H2	110.6306	C17—C16—H16b	109.4715
C3—C2—C7	110.74 (14)	H16a—C16—H16b	106.6803
C3—C2—H2	116.1453	C16—C17—H17a	109.4706
C7—C2—H2	101.7775	C16—C17—H17b	109.471
N2—C3—C2	100.86 (13)	C16—C17—H17c	109.4713
N2—C3—C4	116.93 (16)	H17a—C17—H17b	109.4711
N2—C3—H3	110.9593	H17a—C17—H17c	109.4713
C2—C3—C4	110.85 (17)	H17b—C17—H17c	109.4722
C2—C3—H3	117.026	N2—C18—C19	111.38 (14)
C4—C3—H3	100.9923	N2—C18—H18a	109.4715
C3—C4—C5	106.90 (17)	N2—C18—H18b	109.4713
C3—C4—H4a	109.4713	C19—C18—H18a	109.4707
C3—C4—H4b	109.4714	C19—C18—H18b	109.4714
C5—C4—H4a	109.4711	H18a—C18—H18b	107.4978
C5—C4—H4b	109.471	C18—C19—C20	121.07 (17)
H4a—C4—H4b	111.9296	C18—C19—C24	120.63 (17)
C4—C5—C6	112.75 (15)	C20—C19—C24	118.26 (15)
C4—C5—H5a	109.4716	C19—C20—C21	121.41 (18)
C4—C5—H5b	109.4711	C19—C20—H20	119.2941
C6—C5—H5a	109.4709	C21—C20—H20	119.2942
C6—C5—H5b	109.4716	C20—C21—C22	119.27 (18)
H5a—C5—H5b	105.9755	C20—C21—C25	118.64 (17)
C5—C6—C7	112.22 (17)	C22—C21—C25	122.08 (15)
C5—C6—H6a	109.4716	C21—C22—C23	120.05 (15)
C5—C6—H6b	109.4709	C21—C22—H22	119.9737
C7—C6—H6a	109.4709	C23—C22—H22	119.9726
C7—C6—H6b	109.4708	C22—C23—C24	120.36 (19)
H6a—C6—H6b	106.5811	C22—C23—H23	119.8209
C2—C7—C6	106.70 (17)	C24—C23—H23	119.8211
C2—C7—H7a	109.4713	O6—C24—C19	121.35 (14)
C2—C7—H7b	109.4711	O6—C24—C23	118.01 (18)
C6—C7—H7a	109.4719	C19—C24—C23	120.64 (19)
C6—C7—H7b	109.4712	O4—C25—O5	123.08 (18)
H7a—C7—H7b	112.1028	O4—C25—C21	125.33 (15)
N1—C8—C9	111.39 (17)	O5—C25—C21	111.58 (16)
N1—C8—H8a	109.4707	O5—C26—C27	106.08 (17)
N1—C8—H8b	109.4706	O5—C26—H26a	109.4715
C9—C8—H8a	109.4718	O5—C26—H26b	109.4715
C9—C8—H8b	109.4714	C27—C26—H26a	109.4715
H8a—C8—H8b	107.4817	C27—C26—H26b	109.4709
C8—C9—C10	122.19 (18)	H26a—C26—H26b	112.6645
C8—C9—C14	119.30 (15)	C26—C27—H27a	109.4704
C10—C9—C14	118.4 (2)	C26—C27—H27b	109.4712
C9—C10—C11	121.07 (19)	C26—C27—H27c	109.4714
C9—C10—H10	119.4657	H27a—C27—H27b	109.4716
C11—C10—H10	119.4663	H27a—C27—H27c	109.4708
C10—C11—C12	119.46 (16)	H27b—C27—H27c	109.4719
			
N1—C2—C3—N2	−45.57 (16)	C20—C21—C25—O4	3.4 (3)
C7—C2—C3—C4	65.6 (2)	C15—O2—C16—C17	−86.5 (2)
C10—C11—C15—O1	0.9 (3)	C25—O5—C26—C27	−178.97 (17)

### Hydrogen-bond geometry (Å, °)

**Table:**

*D*—H···*A*	*D*—H	H···*A*	*D*···*A*	*D*—H···*A*
O3—H3o···N1	0.89 (3)	1.82 (3)	2.663 (2)	156.9 (19)
O6—H6o···N2	0.91 (2)	1.82 (3)	2.669 (2)	154 (3)
C2—H2···O1^i^	0.96	2.58	3.362 (2)	138
C3—H3···O4^ii^	0.96	2.57	3.436 (2)	151
C8—H8b···O1^i^	0.96	2.57	3.336 (2)	137
C22—H22···O3^iii^	0.96	2.44	3.351 (2)	159

Symmetry codes: (i) −*x*+1, −*y*+1, −*z*; (ii) −*x*+1, −*y*+1, −*z*+1; (iii) *x*+1, *y*−1, *z*.

## References

[bb1] Agilent (2010). *CrysAlis PRO* Agilent Technologies, Yarnton, England.

[bb2] Bernstein, J., Davis, R. E., Shimoni, L. & Chang, N. L. (1995). *Angew. Chem. Int. Ed. Engl.* **34**, 1555–1573.

[bb3] Brandenburg, K. & Putz, H. (2005). *DIAMOND* Crystal Impact, Bonn, Germany.

[bb4] Burla, M. C., Camalli, M., Carrozzini, B., Cascarano, G. L., Giacovazzo, C., Polidori, G. & Spagna, R. (2003). *J. Appl. Cryst.* **36**, 1103.

[bb5] Cremer, D. & Pople, J. A. (1975). *J. Am. Chem. Soc.* **97**, 1354–1358.

[bb6] Geise, H. J., Buys, H. R. & Mijlhoff, F. C. (1971). *J. Mol. Struct.* **9**, 447–454.

[bb7] Murray-Rust, P. & Riddell, F. G. (1975). *Can. J. Chem.* **53**, 1933–1935.

[bb8] Petříček, V., Dušek, M. & Palatinus, L. (2006). *JANA2006* Institute of Physics, Praha, Czech Republic.

[bb9] Rivera, A., Quiroga, D., Ríos-Motta, J., Dušek, M. & Fejfarová, K. (2010). *Acta Cryst.* E**66**, o931. 10.1107/S1600536810009918 PMC2983906 21580741

[bb10] Rivera, A., Quiroga, D., Ríos-Motta, J., Fejfarová, K. & Dušek, M. (2011*a*). *Acta Cryst.* E**67**, o2627–o2628. 10.1107/S1600536811036385 PMC3201437 22065379

[bb11] Rivera, A., Quiroga, D., Ríos-Motta, J., Fejfarová, K. & Dušek, M. (2011*b*). *Acta Cryst.* E**67**, o2297. 10.1107/S1600536811031205 PMC3200585 22058934

[bb12] Van den Enden, L. & Geise, H. J. (1981). *J. Mol. Struct.* **74**, 309–320.

